# Supplementary material for: Are carbon emissions trading and green financial instruments synergistic? -Comprehensive quantitative research based on content analysis
Source: PLoS One. 2024 Mar 7;19(3):e0298601. doi: 10.1371/journal.pone.0298601 (PMC10919601; doi:10.1371/journal.pone.0298601)
Supplement: S1 Data — (ZIP) [file pone.0298601.s001.zip › Data/地级市Z_F工作报告.zip!/░▓╗╒/│n╓▌╩╨/└┤╫╘╣1⁄2╓┌║┼í╛┬φ┐╦╩2╛▌═°í┐_2018.docx]

各位代表：

现在，我代表市人民政府，向大会作工作报告，请予审议，并请市政协委员和其他列席人员提出意见。

**一、五年来的主要成就**

过去五年，是滁州发展历程中充满挑战、极不平凡的五年。面对错综复杂的内外环境、艰巨繁重的改革发展稳定任务，我们以习近平新时代中国特色社会主义思想为指导，在省委、省政府和市委的坚强领导下，在市人大、市政协的监督和支持下，勠力同心、砥砺前行，全面做好稳增长、促改革、调结构、惠民生、防风险各项工作，较好完成各项目标任务，经济社会继续保持良好发展态势。

——五年来，我们保持定力、精准发力，经济总量冲进全省第一方阵。坚持以新发展理念统揽全局，全面贯彻中央和省各项决策部署，一心一意谋发展，一着不让抓落实。地区生产总值持续上台阶，预计由2012年的971亿元增至1620亿元左右，年均增长9.8%；规上工业增加值总量从全省第9位跃升至第5位，年均增长11.7%；固定资产投资总量实现翻番，年均增长17%；财政收入从153亿元增至289亿元，总量前移3位，跃居全省第四，年均增长13.6%；社会消费品零售总额从313亿元增至575亿元，年均增长12.9%。2014年以来，连续三年获省目标考核先进单位，经济总量如期如愿冲进并站稳全省第一方阵。

——五年来，我们突出重点、破除难点，多项改革走在全省乃至全国前列。弘扬“大包干”精神，突出先行先试，共完成603项改革。勇于创新抓改革。一批改革在全省率先突破，率先完成农村土地承包经营权确权登记颁证试点，率先推出“农权贷”，率先开展政府购买农业公益性服务机制创新，率先开展县级公立医院综合改革，率先推行投资项目“多评合一”改革，率先实现公共资源交易平台市县一体化等；全省第一份农村土地承包经营权证书、不动产权登记证、“五证合一、一照一码”营业执照等在滁颁发。一批改革在全国全省形成品牌，共承担国家、省级试点54项，县域医改“天长模式”、小型农田水利建管机制改革“定远模式”、农村垃圾治理“来安模式”等一批改革经验在全国推广；公车改革、农村环卫治理体制改革等一批全国全省现场会在滁召开。聚力攻坚“去降补”。优化结构去产能，压减60万吨水泥产能。稳定市场去库存，商品住宅库存面积减少226万平方米，去化周期缩短至5.7个月。靶向施策去杠杆，多渠道扩大直接融资，开润股份成功上市，扬子地板、浩淼科技、金春股份、龙利得、天大环保报会待审，新增“新三板”挂牌企业23家；直接融资从18亿元增至110亿元，债券累计发行26只。多措并举降成本，打出“组合拳”，共为企业减负68亿元。突出重点补短板，一批创新平台、基础设施和公共服务项目加快推进。

——五年来，我们扭住中心、把握重心，转型升级迈出重大步伐。着力推动新旧动能转换和结构优化升级，质量效益不断提升，三次产业结构由19.9:52.3:27.8调整为13.9:51:35.1。以创新驱动助推产业升级。国家级创新型试点城市扎实推进，中国家电研究院安徽分院、国家电子元器件质检中心等加快建设，新增国家级企业技术中心2家，建成国家级科技孵化器2家。新建国家级众创空间3家、“星创天地”4家，清华大学启迪之星等一批知名孵化机构建成运营。天长、全椒成为国家知识产权强县工程示范县，明光列为试点县。以育大培强加快工业振兴。工业对经济增长的贡献率达48%，主导地位进一步凸显。统筹推进“三重一创”，业已形成先进装备、智能家电、绿色食品、新型化工、硅基材料、新能源六大支柱产业，其中先进装备产业产值超千亿。实施“三上”工程，狠抓“千亿技改”，开展“四送一服”双千工程，规上企业数翻番、达1615户，康佳电子率先突破百亿大关。建筑业产值增长50%，新增1家特级资质建筑企业。以新型业态提升服务业。加快发展电子商务、文化创意、现代金融、旅游休闲等现代服务业，滁州1912、长城影视城、花博园、苏宁广场、中垦流通、广大物流园等一批项目投入运营。建成电商产业园10个、入驻企业400家，获评中国电商百佳城市。省级服务业集聚区发展到11个，居全省第五。新引进5家市外银行，改制设立4家农商行，村镇银行实现县域全覆盖。3A级以上景区达32家。以工业理念发展现代农业。粮食生产能力达到450万吨，实现“十四连丰”，商品粮收购量稳居全省第一。新增规上农产品加工企业116家。招引培育了辉隆、荣鸿等一批示范项目，现代农业示范区发展到112家，其中国家级1家、省级8家。土地流转总面积460万亩，流转率44%。农机化作业水平、家庭农场和农民专业合作社数量等均居全省前列。

——五年来，我们多措并举、多点发力，重点项目建设取得突破。坚持经济工作项目化、项目工作责任化，落实“四督四保”，持续开展“三看三比”，项目推进步伐加快。招商引资成效显著。始终秉持亲商安商富商理念，一批大项目、好项目成功落户。五年累计招引亿元以上项目1900多个，平均投资规模增长40%，主导产业比重提升13个百分点。亿元以上省外投资项目到位资金2500亿元，居全省前列；实际利用外商直接投资12.2亿美元，年均增长18.6%。重点项目提质提效。五年累计实施亿元以上重点项目1400个，完成投资4300亿元。立讯精密、胜华波汽配、长久轿运车、华塑盐化、奥特佳压缩机、福莱特玻璃等一批项目建成投产。猎豹汽车、天大集团、天康集团、金禾实业、中普能源、长电科技、康佳电子、养元饮品、博西华、滁州卷烟厂等获评全市工业十强。园区建设扩容升级。14个省级以上园区建成区面积扩展到185平方公里，主要经济指标实现翻番。以“一园三城”为主体的国家级产城融合示范区获批建设，西部大工业基地完成规划编研。市经开区经营收入突破千亿大关，规上企业产值占全市1/5，获批中国食品产业基地和省战新产业智能家电集聚发展基地。苏滁产业园一期12平方公里实现“九通一平”，完成投资超百亿，落户企业超百家。天长经开区获批省级高新区。区域合作纵深推进。深度融入长三角城市群和南京、合肥都市圈，全面对接南京江北新区，双圈互动、左右逢源的区域中心城市格局基本形成。凤阳、定远、明光列入皖北振兴政策扶持范围。滁州海关、出入境检验检疫局开关开检，天长海关监管中心和安康公用型、蓝德自用型保税仓库建成运营，恒欣保税仓库获批建设。外贸进出口27.8亿美元，年均增长12.7%，获评全国外贸百强城市。

——五年来，我们精心规划、精致建设，城乡面貌焕然一新。以人的城镇化为核心，着力推进城乡区域协调发展。城镇化率达52%，年均提高1.4个百分点。大滁城再展新貌。更加注重规划引领，新一轮城市总规获批，30余项专项规划批准实施，主城区面积由50平方公里扩展到90平方公里，构建了“141”城市空间，“三区四线”管控力度进一步加大，城市风貌特色进一步彰显。更加注重内涵提升，以一山一河一城一湖“四个一”工程为龙头，加快推进中心城区能级提升，修建了洪武路、西涧路、苏滁大道等30多条主干道，完成了清流路高架、琅狮大桥等一批重点工程，实施南湖、内城河等综合整治，完成小街小巷改造150余处。五年实施重点项目近500个，完成投资230亿元。更加注重城市管理，网格化全面推开，三小车整治和烟花爆竹限放取得实效，成功创建省级文明城市。组团内协调发展，来安“两城同建”“两区并进”，全椒城北新城、城东片区初具规模，琅琊新区、南谯新城建设提速。县城和镇村建设扎实推进。累计实施重点项目2000多个，完成投资超500亿元。天长成功创建全国文明城市，明光揽山引湖入城，定远加快向泉坞山、曲阳湖方向发展，凤阳规划建设淮滨新区。乌衣、汊河、炉桥、板桥、秦栏、大墅等镇容镇貌有了崭新变化。沙河、女山湖等12镇入选全国重点镇。开展“百镇治理”，分层推进中心村建设，天长、全椒等5县市获评全省美丽乡村建设先进县，小岗、黄栗树、小李庄入选农业部美丽乡村创建试点。基础设施全面加强。滁马、徐明高速、宁洛高速凤阳支线、宿扬高速天长段建成通车，滁州港开港通航。滁河防洪治理近期工程完工，江巷水库、淮干治理等进展顺利。建成农村道路畅通工程5400公里。五年完成交通投资500亿元、水利投资63亿元、能源投资180亿元、市政公用设施投资700亿元，水利和市政公用设施投资连续多年居全省前两位。

——五年来，我们铁腕整治、铁面问责，生态文明建设深入推进。绿水青山就是金山银山的理念已成为全社会共识和行动自觉。强化环境治理。开展城市扬尘整治，推进秸秆禁烧和综合利用，淘汰黄标车1.8万辆、燃煤小锅炉700多台，PM10年均浓度值下降18.6%；强化水环境治理，滁河、来河、白塔河、池河等水质总体改善，饮用水源水质稳定达标，西涧湖二级保护区整治成为全国样板；土壤污染防治稳步推进。严格落实中央和省环保督察交办问题整改任务，追责问责近百人。全面完成节能减排目标任务，单位地区生产总值能耗较2012年下降21%。打造绿色家园。百万亩森林增长工程圆满收官，绿色滁州等系列行动深入推进，新增造林150万亩，新增城市绿化2300万平方米，森林覆盖率净增7.6个百分点，达32.5%。成功创建省级森林城市5个、现代林业示范区6个，获评国家园林城市、中国生态文明价值城市。加强生态保护。完成永久基本农田划定，实施矿山地质环境治理项目10个，关闭矿山25处。完成湿地保护总体规划编制，池杉湖湿地公园荣膺国家级试点，女山湖、池杉湖列入省级重要湿地名录。全椒获批全国水生态文明建设试点县，半塔、汊河入选全国生态文明先进镇，舜山、永丰成功创建国家级生态乡镇。琅琊山入选首批“中国森林氧吧”。

——五年来，我们顺应民意、关注民生，人民群众获得感、幸福感、安全感大幅提升。连续实施33项民生工程，认真办好为民十件实事。民生投入持续加大。累计完成民生投入1322亿元，年均增长11.8%。预计城镇、农村居民人均可支配收入分别达到28573元、11942元，年均增长9.3%、10.6%。新增城镇就业44万人。建设保障性安居工程14.5万套，改造农村危房8.2万户。脱贫攻坚取得阶段性成果，16.3万贫困人口稳定脱贫，100个贫困村出列，贫困发生率从5.69%下降到1.1%；定远金融扶贫在全国作经验交流。社会事业全面进步。校园安全工作在全国作经验交流。学前三年毛入园率、高中阶段毛入学率分别达92.7%、98.6%。安徽科技学院、滁州学院办学水平跃居全省地方应用型高水平大学前列。职业技术学院获评全省首批校企合作示范基地，城市职业学院新校区投入使用。建成文化馆、图书馆、博物馆、科技馆19个，基层综合性文化服务中心112个，农家书屋建设成为全国先进。天长、明光、南谯获评全国文化先进县，凤阳、全椒荣膺“中国民间文化艺术之乡”。成功举办第五、六届中国农民歌会。全民健身运动蓬勃开展，举办了首届国际半程马拉松赛和首届残疾人运动会，天长等成为全国群众体育先进单位。市一院南院区建成运营，南京儿童医院滁州分院正式启用，基层卫生院和社区卫生服务机构标准化建设全面完成，基本公共卫生服务水平不断提升。荣获全国科技进步先进市“两连冠”、双拥模范城“五连冠”、人民防空先进城市等称号，防震减灾工作跻身全国前十，法律援助、无偿献血等工作受到国家表彰。和谐局面更加稳固。建立“乡为主”信访工作机制，在全省率先探索实行联合接访、阳光信访、专题约访等制度。深化平安滁州建设，推进“六六联创”工程，强化治安防控和公共安全体系建设，人民调解中心全椒经验在全省推广。突出重点行业领域安全监管，安全生产形势持续平稳。红十字会、慈善和残疾人等事业取得新成绩，工会、共青团、妇联、文联、科协等群团组织作用进一步发挥，编制、供销、物价、对台、保密、档案、地方志、工商联、外事侨务、民族宗教、关心下一代等工作全面进步。

——五年来，我们依法行政、从严治政，自身建设持续加强。恪守“为民、务实、清廉”，驰而不息转职能、改作风、优服务。切实强化“四个意识”。党的群众路线教育实践活动、“三严三实”专题教育、“两学一做”学习教育取得重要成果，“讲看齐、见行动”学习讨论、“讲重作”专题教育和专题警示教育扎实开展。始终把纪律和规矩挺在前面，坚决维护党中央权威和集中统一领导。着力规范行政行为。自觉接受人大、政协和纪检、司法、社会各界监督，主动听取民主党派、工商联、无党派人士和人民团体意见建议，五年共办结人大议案17件、建议610件和政协提案1876件，满意率不断提高。推进依法科学民主决策，加强规范性文件审查管理，实现重大事项合法性审查全覆盖。建立健全“3+2”清单制度体系，推进“双随机一公开”，加强政府信息公开、舆情回应和政策解读，开展基层政务公开标准化规范化试点，让权力在阳光下运行。认真落实“一岗双责”。持之以恒落实中央八项规定精神和省、市有关规定，“四风”突出问题有效遏制。深入推进政府系统党风廉政建设和反腐败斗争，强化行政监察和审计监督，从严查处违纪案件。政风建设考核2016年跃居全省第一。

**二、过去一年的工作**

各位代表，五年的成就振奋人心，去年的工作尤为给力。这一年，我们聚焦重点，聚力落实，经济社会发展迈上新台阶。着力抓了以下七个方面：

（一）坚持高点定位，经济发展再创新业绩。紧紧围绕冲刺总量全省第三目标，结合实际，精心谋划，市委市政府先后召开全市创新发展、制造业发展和旅游业发展三个牵动性强的大会，明思路、定措施、抓落实，有力推动经济社会持续健康发展。总量指标中，预计地区生产总值、规上工业增加值、固定资产投资、财政收入、外贸进出口、实际利用外商直接投资6项指标稳居全省第一方阵；动态指标中，预计地区生产总值、规上工业增加值、固定资产投资、财政收入、外贸进出口、城镇居民人均可支配收入6项指标均居全省前5位。

（二）突出创新驱动，转型升级取得新成效。围绕创建国家级创新型城市目标，外学苏浙沪、内学合芜蚌、主抓“六个一”，着力下好创新“先手棋”。建立“1+4+19”科技创新政策体系，开展院士助滁、引博行动，全年新增院士工作站10家，居全省第二；新引进两院院士10名、博士等高层次人才160多名；4个创新团队获省高层次科技人才团队认定，居全省第四。新增省级以上研发平台24家，总数达151家，居全省第五。发明专利授权550件，居全省第四。工业上，六大支柱产业产值增长15%，战新产业产值增长27.5%；高新技术企业净增56家、总数达286家，高新技术产业实现产值超1600亿元，居全省第三。我市获批国家级消费品工业“三品”战略示范市。全柴动力、康佳电子获批国家智能制造专项。“四送一服”双千工程年度考核居全省第一。服务业上，电子商务发展提速，与阿里巴巴、京东、苏宁等合作深入开展，提前实现电子商务进农村全覆盖，网上交易额超320亿元，其中网萌、云伙计分别达180亿元、110亿元。树立“大旅游”理念，积极打造特色旅游板块、旅游线路和旅游产品，推动景点旅游向全域旅游转变。外贸进出口全年保持双增长态势，创历史最好水平。新增贷款超270亿元，增长19%。浦发银行、光大银行开业。农业上，稳步推进现代农业发展，加快构建现代农业产业体系、生产体系、经营体系。规上农产品加工企业发展到344家，实现产值610亿元，增长10%。家庭农场6500多家、农民专业合作社6200多个。明光、全椒被认定为全国农业农村信息化示范基地，天长成为全国主要农作物生产全程机械化示范县。“三品一标”认证企业数全省第一。

（三）狠抓先行先试，多项改革创出新品牌。坚持抓小岗促全市，重点领域和关键环节改革取得新突破。县域医改“天长模式”和家庭医生签约服务“定远模式”得到中央肯定，全国县级公立医院综合改革示范工作现场会在天长召开，“学天长、全覆盖、再创新”在全市推开；全国家庭医生签约服务经验交流会在定远召开。率先开展医保管理体制改革试点，“三保合一”机构设置全面完成。农村综合改革深入推进，天长农村集体资产股份权能改革全国领先，全国总结交流会在天长举行；凤阳小岗、来安罗庄的省级“三变”改革试点顺利完成。“放管服”改革持续深化，在全省率先开展“减证便民”行动，政务服务标准化国家级试点中期评估顺利通过，基层政务公开全省现场会在定远召开，实现“一张网”省市平台对接。在全省率先完成城管体制、国有林场等改革任务。顺利完成市自来水公司股权改革。深入开展小岗“三年大提升”行动，小岗农村宅基地和农房确权发证稳步推进，农民合作社、家庭农场风险补偿基金试点取得实效，盼盼食品等项目开工建设。

（四）紧盯重点项目，招大引强实现新突破。高位推动、高频调度，组建60个招商小分队，全年亿元以上项目新签约440个、新竣工310个、新达产115个。加速招商成果转化，总投资200亿元的兴茂国际旅游、100亿元的荣盛湿地旅游、60亿元的国能锂电池、50亿元的亚玛顿等一批重大项目相继落户。特别是总投资240亿元的惠科项目，是滁州有史以来单体投资规模最大的工业项目，从签约到开工仅用了57天时间，创造了“滁州速度”。

（五）推进城乡统筹，人居环境有了新提升。高度重视城乡规划，以建设省级“多规合一”试点市为契机，基本建立了区域规划、总体规划、详细规划相互衔接的规划体系，形成了覆盖全市的空间管控“一张图”。统筹推进城乡建设，大滁城竣工项目77个、在建118个，完成投资近百亿元。“四个一”工程加快推进，琅琊山创5A提速提效，北大门改造竣工，龙池街一期完工，太仆寺展露新姿，花博园开园迎客；清流河一期提升即将完成，二期建设完工，三期完成施工图设计，四期开工建设；滁州古城保护性改造有序进行，琅琊路、西涧路、清流路提升工程完成，遵阳街、清流街、北湖等改造加快实施；明湖正式下闸蓄水，路网工程快速推进，景观工程全面展开。同时，各县城打造一批城市建设精品项目，一批特色小镇和美丽乡村建设扎实推进，汊河入选全国特色小镇，小岗获评“中国十大最美乡村”。基础设施进一步完善，滁淮高速、合宁“四改八”加速推进，滁州大道分段建设，滁西环高速即将开工，滁宁城际（滁州段）工可报告通过专家评审，合新、合滁宁高铁、宁淮城际和滁天、来六、明巢高速等前期工作加快推进。滁州机场场址初步选定。

（六）树牢绿色理念，生态建设取得新进展。把生态文明建设摆上更加重要的战略位置，坚决打好大气、水、土壤污染防治三大战役，切实抓好中央和省环保督察反馈问题整改。圆满完成PM2.5年度目标任务，PM10控制幅度居全省第二。《滁州市市区饮用水水源保护条例》颁布实施，全域推行河长制，设立河长3400多名，7个国家考核断面水质全部达标。滁城第三污水处理厂建成，城东污水处理厂扩容项目完成主体工程。扎实推进“林长制”，新增造林21万亩，老嘉山成为国家级森林公园。推动绿色循环低碳发展，凤阳国家“城市矿产”示范基地通过验收。扎实开展农村环境“三大革命”，实现农村垃圾治理全覆盖，来安、全椒被纳入国家生活垃圾分类和资源利用示范县，完成55个乡镇政府驻地和31个省级中心村污水设施建设。

（七）着力改善民生，和谐发展呈现新局面。全力推进各项民生工程，让发展成果更多更好地惠及群众。脱贫攻坚成效显著。以产业扶贫为重点的十大工程出新出彩，落实产业扶贫到户项目7.1万个，实施健康脱贫“351”“180”政策，贫困户子女从学前教育到大学阶段实现资助全覆盖。改造贫困户危房1.1万户。44个贫困村出列、4.6万名贫困人口脱贫。全省扶贫小额信贷现场会、旅游扶贫现场会分别在定远、凤阳召开。社会事业协调发展。新建、改扩建公办幼儿园21所，新增普惠性民办幼儿园53所；提前一年实现县域义务教育基本均衡全覆盖目标。建立分级诊疗制度，县域就诊率稳步提升，乡镇门诊达72万人次。全面落实两孩政策。公共文化服务体系不断完善，市科技馆、博物馆开馆，明中都皇故城成为全省首个国家考古遗址公园，新增全椒八朵云等5项省级非遗项目。新增5个全国文明单位、3个全国文明村镇。7个县市区被命名为全省平安县，小岗义务调解工作成为全国样板。定远“救急难”在全国作经验交流。开通公交“亭城通”，实现与南京公交互换互乘。十件实事扎实推进。超额完成2000户滁城棚户区改造任务；完成4个老旧小区改造和20个安置小区配套工程；建成街头游园10个；新建城南新区公厕5座；完成10个小区二次供水改造；完成4个社区公共服务用房达标工程；完成10处人行道、盲道完善工程；建成8处流动摊点集中便民服务点；完成8条城市主干道亮化复绿工程；花鸟鱼虫市场迁建项目主体完工。

各位代表！过去一年取得的丰硕成果，为本届政府工作画上了圆满句号。

五年来，我们深入落实习近平总书记系列重要讲话特别是视察安徽、亲临小岗重要讲话精神，直面困难和挑战，不动摇、不懈怠、不折腾，办成了一些对滁州未来发展具有积极而长远影响的大事，使这五年成为滁州综合实力提升最快、城乡面貌变化最大、群众获得感最强、市内外公众评价最好的时期之一。

成绩来之不易，成绩十分珍贵！这是省委、省政府高度重视、亲切关怀的结果，是市委总揽全局、坚强领导的结果，是市人大、政协和社会各界鼎力支持、悉心帮助的结果，更是全市人民奋力拼搏、锐意进取的结果。在此，我代表市人民政府，向全市人民，向人大代表、政协委员和党代会代表，向各民主党派、工商联、无党派人士和各人民团体，向中央及省驻滁单位，向人民解放军、武警官兵、公安政法干警，向所有关心、支持、参与滁州建设与发展的各位老领导、老同志和各位客商、各界朋友，表示衷心的感谢，并致以崇高的敬意!

回首五年，我们深切感受到，办好滁州的事情，必须坚持党的领导，坚决维护党中央权威和集中统一领导，立场坚定、态度坚决，步调一致跟党走。必须坚持解放思想，与时俱进，开拓创新，以新理念引领新思路，以新举措推动新发展。必须坚持发展不动摇，主动适应、把握、引领新常态，厚植发展新优势，闯出发展新路子。必须坚持以人民为中心，把人民对美好生活的向往作为奋斗目标，让发展成果更多更公平地惠及人民，让人民群众在共建共享中拥有更多获得感。必须坚持改革创新，传承改革基因，弘扬敢为人先的“大包干”精神，不断擦亮改革品牌，持续激发创新创业创造活力。必须坚持维护稳定大局，最大限度增加和谐的正能量，最大限度减少影响稳定的因素，倍加珍惜心齐气顺、风正劲足的大好局面，持续营造干部干净干事、群众群策群力的良好政治生态，为全面建成小康社会提供有力保障。这“六个必须”，是我们过去五年积累的宝贵经验，是我们开启新征程、开创新局面的信心和底气，是我们未来发展的不竭动力源泉，激励着我们迎难而上、勇往直前。

站在新的起点上，我们更要保持清醒，认真审视前进中的困难和问题。主要是：发展不足、发展不优、发展不协调仍然是现阶段我们面临的最大问题；一些重点领域和关键环节的改革突破不够，创新能力不强，新动能尚未真正形成，转型升级任重道远，资源环境等瓶颈制约加剧；实体经济发展还面临用工紧张、融资不畅、成本升高、服务不到位等不少困难；县域经济总体较弱、发展还不平衡；民生领域还有不少短板，脱贫攻坚任务艰巨，教育、医疗、住房、养老等领域的优质资源供需矛盾仍很突出；市民文明素质和社会文明水平尚需提高；社会矛盾和问题交织叠加，治理能力有待加强；少数政府部门和工作人员不敢担当、不愿作为，一些领域的消极腐败现象仍然易发多发等。这些问题，必须着力加以解决。

**三、未来五年的目标任务**

党的十九大作出中国特色社会主义进入新时代的战略判断，明确了从现在起到本世纪中叶“两步走”的战略安排，描绘了建成社会主义现代化强国的美好蓝图。省委十届六次全会明确提出了“三个同步”的阶段性目标。市委六届五次全会对滁州今后一个时期的目标任务作出安排。总体目标是，与全国全省保持“三个同步”，即：同步全面建成小康社会，同步基本实现社会主义现代化，同步建成社会主义现代化强市。阶段安排是，把握好五个节点：到2018年末，主要经济指标增幅全省靠前，总量进位或缩小与排位在前城市的差距；到2020年末，全面建成小康社会，地区生产总值、财政收入总量冲进全省第三；基本实现市第六次党代会确立的“346”目标；到2022年末，多数经济指标总量进入全省前三，人均指标超全省平均水平；到2035年，综合实力进入省内强市行列，基本实现现代化；到2050年，全面建成富强民主文明和谐美丽的现代化新滁州。

未来五年，我们既要决胜全面建成小康社会，又要乘势而上开启滁州现代化建设新征程。综合分析各方面因素，滁州仍处于大有可为的重要战略机遇期。我们必须准确把握滁州发展阶段性特征和新形势、新任务、新要求，坚持战略思维、创新思维、辩证思维、法治思维、底线思维，保持定力，主动作为，以更高的站位、更严的要求、更实的举措、更硬的作风，做好各项工作，奋力把滁州发展推向新的更高水平。

今后五年工作的指导思想是：全面贯彻党的十九大精神，以习近平新时代中国特色社会主义思想为指导，坚持以人民为中心，坚持新发展理念，坚持高质量发展，统筹推进“五位一体”总体布局，协调推进“四个全面”战略布局，按照“三个同步”和“五个阶段”的安排，聚焦“346”奋斗目标，以供给侧结构性改革为主线，以五大发展行动计划为总抓手，坚持稳中求进工作总基调，好中加快、能快则快，加快建设现代化新滁州。

——综合实力再上新台阶，多数指标进入全省前三。经济保持中高速增长，主要指标增速全省争先，人均指标超过全省平均水平。产业迈向中高端，加快三次产业结构由“二三一”向“三二一”转变。以“五个一号工程”为引领，推动发展质量和效益实现双提升，基本形成创新驱动发展新格局。高新技术产业产值突破3000亿元。

——城乡统筹达到新水平，基本实现公共服务均等化。坚持“一尊重五统筹”，全力推进“四创一建”，滁城首位度进一步提高，辐射作用显著提升，县域经济实力持续增强。以大滁城为主体、县城为支撑、重点镇为节点、美丽乡村为基础的城乡体系基本形成。交通网络互联互通，基础设施进一步夯实。基本公共服务覆盖面持续扩大。常住人口城镇化率达到59%，跻身全国文明城市行列。

——生态环境获得新改善，绿色发展走在全省前列。坚持生态效益与经济效益、社会效益相结合，加快形成低碳生产、生活方式，资源节约集约利用取得重要进展。打赢大气、水、土壤污染防治三大战役，生态质量显著提升。森林覆盖率达36.2%以上，城市绿化覆盖率提升到43%，中心村环境整治达标率达100%。

——民生福祉实现新提升，全面建成小康社会。坚决打赢脱贫攻坚战，现行标准下农村贫困人口全部脱贫。城乡居民收入增速高于经济增长，收入差距逐步缩小。社会就业更高质量、更加充分，社会分配更加公平、更加合理。健全社会保障体系，强化城乡最低生活保障，完善社会福利和社会救助，实现社会保障全覆盖、更公平、可持续。着力防范化解重大风险，推进治理体系和治理能力现代化，群众获得感、幸福感、安全感继续提升。

实现上述目标，要坚持远谋近做，以“五个一号工程”为重点推进五大发展，以“五大行动”为重点推进补短板、促平衡，以“五大产业”为重点推进供给侧结构性改革，以“五大改革”为重点推进“抓小岗促全市”，以“五大战役”为重点推进防风险、强弱项。在此基础上，立足于高质量发展，着力聚焦“五个方面”：

（一）聚焦创新创业，着力强化产业支撑。推动经济发展质量变革、效率变革、动力变革，提高全要素生产率。加快建设国家级创新型试点城市，积极培育原始创新，重点突破集成创新，全面提升引进消化吸收再创新。建设智能制造强市，深入实施《中国制造2025》，加速推进“三重一创”建设，大力改造提升传统产业，加快大数据、云计算、物联网应用。以惠科项目为创新发展“一号工程”，重点发展先进制造、特色农业、文旅康养、智慧经济、现代金融等五大产业，加快产业升级。培育10家产值超百亿企业，打造智能家电、新能源汽车及装备制造、健康营养、非金属矿精深加工4个千亿产业，推动市经开区经营收入五年翻一番，滁州高新区经营收入超千亿，西部大工业基地力争到2020年产值超千亿。琅琊山、小岗村成功创建5A级景区，现代服务业水平加快提升。

（二）聚焦协调协同，着力建设美丽家园。以东部国家级产城融合示范区和西部大工业基地建设为协调发展“一号工程”，突出产城融合和城乡一体，持续提升城市品质。加快与南京同城化、合肥一体化进程，着力打造省际交汇区域中心城市。统筹推进大滁城、县城、特色小镇和美丽乡村建设，推进区划调整，促进城乡融合发展。实施乡村振兴战略，坚持农业农村优先发展，加快建设产业兴旺、生态宜居、乡风文明、治理有效、生活富裕的新农村。

（三）聚焦绿水青山，着力改善生态环境。绿水青山是大自然赐予滁州的宝贵财富，是我们永续发展的根本保障。以明湖生态湿地建设为绿色发展“一号工程”，着力打造生态明湖、智慧明湖、传世明湖。加快省级绿色生态城市综合试点建设，争创国家级生态文明先行示范区和生态市、森林城市。统筹山水林田湖草系统治理，深入实施大气、水、土壤污染防治行动计划，实行最严格的生态环境保护制度，形成绿色发展方式和生活方式，走出一条经济发展与环境改善双赢之路。深入开展绿色滁州行动，围绕“一核两轴三网四级多点”生态空间和产业布局，重点发展“三树一苗”150万亩以上，聚力发展七大林业产业。全面促进资源节约集约利用，让城乡环境更加优美。

（四）聚焦动力活力，着力深化改革开放。始终把改革作为推动发展的关键一招，争当改革先锋。聚力抓好农村综合改革、综合医改、放管服、新型城镇化、社会事业领域等五大改革，最大限度激发活力、释放红利。积极融入“一带一路”和长江经济带，统筹对内合作与对外开放，加强与长三角、珠三角等地区的交流合作，深度融入南京、合肥都市圈，形成全方位开放格局。以滁宁城际铁路建设为开放发展“一号工程”，建设大江北协同区。

（五）聚焦共建共享，着力增进人民福祉。以造福人民为最大政绩，想群众之所想，急群众之所急，在“七有”上不断取得新进展，让全体人民在共建共享中有更多获得感。以脱贫攻坚为共享发展“一号工程”，坚决打赢脱贫攻坚战，确保小康路上一个都不能掉队。坚持教育优先发展，实施“健康滁州2030”行动，健全覆盖城乡的基本公共服务体系。争创全国文明城市，争办第十五届省运会。着力提高城乡居民收入，推动更高质量的就业。建成覆盖城乡居民的社会保障体系，均衡发展居家养老、社区养老、机构养老、医养结合等多样化养老服务。建设平安滁州，创新社会治理，加强生产安全、农产品和食品药品安全，维护社会和谐稳定。

**四、2018年重点工作安排**

2018年是贯彻党的十九大精神的开局之年，是改革开放40周年，是决胜全面建成小康社会、实施“十三五”规划承上启下的关键一年，也是新一届政府的起步之年。做好今年各项工作，意义十分重大。

经济社会发展的主要预期目标是：地区生产总值增长8.5%以上，规上工业增加值增长9%以上，固定资产投资增长12%，财政收入增长9%以上，社会消费品零售总额增长10%以上，城镇和农村居民人均可支配收入增速高于经济增长，城镇登记失业率控制在4.4%以内，完成节能减排任务。在实际工作中，全市要确保更快（增幅位次保证在全省前五），各县市区要务求多超（增幅在全市平均水平以上）。

重点做好八个方面工作：

（一）以创新发展为引领，加快新旧动能转换。

打造创新平台。推进产业技术创新、区域协同创新、大众创业万众创新。加快以“一园三城”为主体的国家级产城融合示范区建设，促进科技资源、人才资源和金融资本向示范区集聚。扩大科技金融风险补偿资金池规模，培育科技成果转化中介服务机构，推动更多科技成果在滁转化。力争新增省级以上研发平台24家，科技孵化器、众创空间5家。

培育创新主体。完善以企业为主体、市场为导向、产学研相结合的技术创新体系，加快培育一批创新型企业。新建省级企业技术中心6家、工业设计中心3家，培育国家级技术创新示范企业1家、省级2家，高新技术企业超300家。引导企业持续加大研发经费投入，力争研发投入强度进入全省前三。扶持企业开展重大科技专项攻关，全年授权发明专利600件以上。争取省级科技奖补类、计划类项目80个以上。全年新增各类市场主体超2.7万户，其中私营企业5000户以上。

聚合创新人才。进一步完善人才培育、引进和使用机制，加快培育本土人才，大力引进市外高端人才。创新人才引进政策，柔性引进“高精尖缺”人才，吸引更多优秀人才在滁创新创业。落实好“1+4+N”政策体系，通过提供住房扶持、薪酬补贴、子女入学、配偶就业、创业空间等方式，不断降低人才创新创业和居住生活成本，使人才引得进、留得住、用得好。

（二）以改革开放为动力，持续厚植发展优势。

更大力度推进改革攻坚。把深化改革作为对改革开放40周年的最重要纪念，坚持抓小岗促全市，进一步擦亮滁州改革品牌。农村综合改革上，争创农业农村改革示范区，积极争取各类涉农改革试点，力争打造具有全国影响力的改革品牌。完善“三权分置”制度，积极探索土地承包权、宅基地使用权和集体收益分配权退出机制。推进“三变”改革试点，激活农村各类生产要素。扩大农村集体产权制度改革试点，实现乡镇全覆盖。创新农业社会化服务体制，开展公益性、经营性、社会化服务创新试点，打通服务三农“最后一公里”。小岗村要充分发挥改革示范引领作用，聚焦农村土地制度、集体产权制度、农业经营制度等，统筹推进，重点突破，努力在中国农村改革历史长河的每个断面都留下深深的滁州印记，为全省乃至全国农业农村改革探索新路子、积累新经验。综合医改上，抓好省级医保管理体制改革试点，完成“三保合一”。深入推进医联体、医共体建设，完善医疗卫生服务体系，进一步破解“看病难、治病贵、防病弱”等突出问题，着力打造医改“滁州模式”。放管服改革上，不断优化权责清单，严格执行涉企收费清单，全面实施市场准入负面清单，推广公共服务清单和行政权力中介服务清单。深化行政审批制度改革，加快政务服务“一张网”建设，推进“最多跑一次”和“不见面审批”改革。深化商事制度改革，加快电子营业执照系统应用，探索全城通办、局所联办、银行代办等便利化登记服务。推进公共资源交易监管体制改革，规范交易行为。新型城镇化改革上，全面放开落户限制，从住房、就业、子女上学、社保等方面研究制定政策措施，增强城镇对农业转移人口的吸引力和承载力。推进住房保障金融服务体制改革，建立健全政府住房租赁交易服务平台，推进惠民住房金融服务公司稳健运营，加强住房公积金建制扩面。经济体制改革上，抓好投融资体制改革，完善PPP模式，鼓励和支持社会资本参与基础设施等重点领域建设。完善园区管理体制，赋予开发区更多自主权。引入非公资本，发展混合所有制经济。引导民企通过挂大靠强、兼并重组、股改上市等途径，加速成长。社会事业改革上，今年突出抓好人力资源改革，着力解决好结构性就业矛盾，针对劳动力不足与劳动力外流、劳动力浪费并存的问题，推出更加积极的就业再就业、返乡就业创业等举措，再造滁州劳动力新优势，为企业增人力、为城市聚人气。同时，统筹推进文化、生态文明等领域改革，不断为发展增添新动力。

更高水平强化开放合作。加快融入“一带一路”和长江经济带，不断提高对外开放水平。深度融入长三角，加快产业、市场、通关等一体化进程。积极推动与江北新区全面对接、等高对接、协同对接，支持汊河、乌衣、金牛湖等节点地区深度融入；推动滁宁合作项目化，促进城市规划、基础设施、产业转移、公共服务等方面共建共享。推进与合肥一体化，深化基础设施、产业发展、要素市场等方面合作。落实省促进皖北发展政策，参与淮河生态经济带建设。启动“无水港”项目，积极开展铁海联运业务，鼓励企业走出去。新增进出口实绩企业100家以上。

（三）以转型升级为主线，着力壮大实体经济。

推动乡村加快振兴。按照“20字”总要求，以农业产业化水平提升、特色精品农业打造等12项工程为着力点，落实具体举措，分类分项推进，力促农业强、农村美、农民富。加快农业供给侧结构性改革，大力发展现代农业，新增1000个家庭农场，争创1个国家级、2个省级和8个市级现代农业产业园，农产品加工业产值增长10%以上。打造特色农业，重点发展优质稻米和小麦，实现粮食总产超86亿斤；因地制宜，加快发展特色杂粮，把“小品种”做成“大产业”。打造绿色农业，大力推广绿色种养模式，力争“稻虾连作”发展到22万亩，争创1个国家级稻渔综合示范区。打造功能农业，依托滁菊等，研发生产大健康食品，大力发展休闲、观光、康养农业及“互联网+”等新型业态，争创省级田园综合体试点，促进“接二连三”发展。打造品牌农业，通过质量提升、标准化生产、特色产品认定等，发展品牌粮油100万亩、特色果蔬茶（菊）50万亩、健康水产50万亩，生产优质畜禽产品30万吨，新增“三品一标”认证产品100个。推动美丽乡村建设再上新水平，持续推进68个省级中心村建设。改造农村危房1万户。推进农村环境“三大革命”，90%以上村庄脏乱差得到有效治理。打好精准脱贫年度战役，既不降低标准，也不吊高胃口，尽锐出战，统筹抓好产业扶贫、就业扶贫、教育扶贫、健康扶贫等，确保实现定远县摘帽、23个贫困村出列、2.9万贫困人口脱贫年度目标。

推动制造业做大做强。坚持工业强市不动摇，大力推进两化融合，加快发展先进制造业，全年新增规上企业超80户。围绕高端制造，抓好“三重一创”，加快“1+8”战新基地建设，实施“三个一百”工程，力争全年实现战新产业产值870亿元，占规上工业产值20%以上。围绕智能制造，深入开展“千亿技改、千企升级”和互联网+制造业行动，力争全年完成技改投资470亿元，培育省级智能工厂、数字化车间和两化融合示范企业各5家。围绕精品制造，加快国家级消费品工业“三品”战略示范市建设，力争全年开发新产品300个以上，培育安徽名牌企业20家、标准化示范企业2家、工业精品6个，省市级专精特新中小企业60家以上。围绕育大培强，推动“龙头+配套”集群发展，力争全年新增百亿元和50亿元企业各1家、亿元企业20家。围绕精准帮扶，常态化开展“四送一服”双千工程，降低制度性交易成本。构建亲清新型政商关系，全力创优“四最”营商环境。

推动服务业提档升级。加快培育文旅康养、智慧经济、现代金融等现代服务业，力争服务业增加值增长11%。以文旅康养产业为突破口，加快发展全域旅游，狠抓“五个一批”工程建设，年度完成总投资50亿元以上；狠抓品牌创建，谋划推进新业态旅游项目10个以上；加快全椒、南谯创建国家全域旅游示范区，不断拓展旅游承载空间。推进京东数字产业园、滁州跨境电商产业园、创达电子商务城建设，推动网萌、云伙计、子曰科技等做大做强，全年电子商务交易额突破380亿元。大力培育限上商贸企业，力争总数超1000家。强化金融服务实体经济导向，防止金融“脱实向虚”，建立健全农业融资担保体系，稳妥处置地方政府债务，打好化解重大风险战役，坚决守住不发生系统性金融风险的底线。力争新增上市企业2家以上，新增贷款250亿元以上，直接融资超120亿元。建立多主体供给、多渠道保障、租购并举的住房制度，促进房地产市场平稳健康发展。

推动县域经济加快发展。围绕做大规模、做强产业、做出特色，促进县域分工协作，实现特色发展、错位发展、竞相发展，在全省考核中务求增速争先进、总量进位次、人均上台阶，全年县市区经济总量达到1550亿元。

（四）以重点项目为支撑，不断扩大有效投入。

聚力双招双引。坚持抓招大引强，聚焦高端、智能、绿色制造，突出新型显示、新能源汽车、大健康等新兴产业，着力引进产业链龙头项目、创新链关键项目和价值链高端项目。紧盯华为、腾讯、小米等知名企业，主攻品牌引入。要以惠科项目为牵引，大力引进上下游配套企业；集聚放大家电板块，再造一个“博西华”，力促康佳产业园启动建设，加快一批项目签约落地，尽早形成千亿智能家电产业。抓好专业招商，组建80个小分队，推进大招商、招大商。力争全年引进市外亿元以上项目超400个，到位资金950亿元，力争百亿级大项目县市区基本覆盖。着力抓招才引智，力争全年新组建院士工作站10家左右，引进高层次人才团队15个以上。

加快项目推进。落实“四督四保”制度，加大重点项目攻坚力度，进一步扩大有效投入。力求平板显示、光伏玻璃等一批重大项目尽快签约落地；新开工建设朝野电视、国能锂电池、三棵树涂料、立光电子、远大装配式建筑产业园等亿元以上项目400个；加快推进隆达电子、迈达电子等400个在建项目；力促天鼎丰非织造布、嘉安汽车、远达彩印等300个项目竣工；谋划储备项目600个以上。加快PPP项目建设，争取入库项目100个以上。

提升园区平台。推进开发区优化整合，坚持“筑巢引凤”，加快“腾笼换鸟”，建成区面积拓展到200平方公里，进一步提升综合承载力，主要指标增速高于全市。市经开区要争先进位，冲刺全国50强；要快速推进原创城建设，尽快形成引领全市的创新高地、高技术产业集聚区。苏滁产业园要拉高标杆，围绕主导产业，确保新签约、新开工、新投产亿元以上工业项目分别达40个、30个、20个以上。引入30亿元以上或3亿美元以上的大项目再有新突破。推动滁州高新区创建国家级高新区，全椒开发区扩区获批。推进凤阳·宁国、定远·肥东、明光·肥西合作园区建设，加快定远盐化工业园建设步伐。

（五）以提升品位为路径，全力建设美丽滁州。

全域化规划。加快区域协同规划、全市域空间规划、城镇总体规划修编等，深化“多规合一”试点，加快构建核心引领、板块联动、多点支撑的区域发展格局。创新规划设计理念，抓好建筑形态、绿地景观、特色街区、地下空间利用的规划设计，促进各要素之间功能相匹配、形态相适应。强化规划管控，坚持项目跟着规划走、资金跟着项目走、监管跟着资金走，促进城市有序建设与发展。

精致化建设。大滁城建设上，以“四个一”工程为重点，实施好总投资330亿元的200个左右重点建设项目，完成投资130亿元以上。琅琊山成功创建5A级景区，启动东门、南门等景区配套工程，实施凤凰湖、清流关、醉心谷、福囍小镇、铜话小镇等文旅项目，连片打造好丰乐亭和太仆寺景区，改造清丰路、览山路、世纪大道延伸段等旅游道路。清流河景观加速提升，精致建设三期、四期工程，加强水环境综合治理。古城风貌基本形成，启动内城河三期，实施紫薇路北延、中都大道北延等工程，推进遵阳街、北湖区域改造和阳明书院建设，完成南谯北路桥等工程，打造金刚巷等历史文化街区，清流街开街运营，完成章益、吴棠故居修缮及布展。明湖建设全面提速，启动45万平方米园林绿化工程，抓好奥体中心、明湖中学、康养小镇、国际社区等公建设施及路网建设；加快推进与苏宁集团等战略合作，谋划实施好“双千工程”。完善城市公共基础设施，实施四水厂三期及第四污水处理厂新建、清流污水厂扩容、公共停车场等项目建设。县城建设上，各县城要坚持产城结合、建管结合、新老结合、城乡结合，进一步完善功能、强化管理、提升水平。天长、明光、定远、凤阳四个县城要拓展新区、提升老城，新建续建项目超400个，完成投资超300亿元。镇村建设上，因地制宜、突出特色，加快水口、炉桥、大墅、临淮关等重点镇建设，精心培育铜城国家级特色小镇和秦栏、女山湖2个省级特色小镇。

网格化管理。全面动员、全民参与，全力开展全国文明城市创建行动，对照创建要求，既统筹兼顾打好总体战，又要聚焦重点难点打好攻坚战，务必完成首战阶段性目标。全面落实“街道主责、社区主抓、城管主力、单元网格主体”的管理体系，提升数字化管理水平，探索快速处置、非现场执法等新型执法模式。大力推进“两治三改”，整治车辆乱停乱放、店外店和占道经营等。优化交通组织，推进城区治堵提速。

一体化完善基础设施。加速推进滁淮高速、合宁“四改八”工程，争取滁宁城际（滁州段）、合新高铁和滁西环、滁天、来六、明巢高速等开工建设，实施好滁州大道、G328绕城段、G104明光段等10条干线工程，做好合滁宁高铁、宁淮城际、滁州机场等项目前期工作。建设美丽公路100公里。完成汊河船闸重建工程，加快江巷水库建设，推进滁河新增治理工程、沙河集水库增蓄工程、黄栗树水库除险加固工程和新建山许水库等前期工作，除险加固小型水库110座，解决7万农村人口安全饮水问题。统筹推进500千伏同乐变、220千伏相官变、110千伏珠江变等电力项目，及中海油油气管线、信息网络等基础设施建设。

（六）以生态治理为重点，倍加呵护碧水蓝天。

培育绿色基因。深入实施绿色滁州行动，加快绿色矿业和绿色矿山建设，落实好林长制，加大河流、道路、农田林带林网建设力度，打造生态屏障和生态景观区。注重绿化管护，推进森林公园建设，改善城市绿地系统。全年完成造林21万亩，创建1个省级森林城市、6个省级森林城镇、40个省级森林村庄。

打好污染防治战役。持续抓好中央和省环保督察反馈问题整改，完成全国第二次污染源普查，推进生态监测网络体系建设。打好蓝天保卫战，突出工业废气、餐饮油烟、城市扬尘等污染治理，加强秸秆禁烧和综合利用；打好碧水攻坚战，深入推进河长制，强化饮用水水源地保护，抓好黑臭水体、农业面源污染等治理；打好净土持久战，全面落实“土十条”，推进化肥农药零增长行动，强化土壤环境综合监管，推进定远、来安2个国家级治理修复试点项目，实施13个市级治理修复项目。坚持铁腕治污，从严治污，严厉打击各类环境违法行为。

推进生态保护。抓好生态空间管控与保护，守住耕地保护、开发强度、生态保护三条红线，更大力度推进节能减排降耗工作。推进园区绿色低碳循环发展，推动产业优化升级和资源集约节约利用。加快矿产资源整合，做实做大兴滁矿业。积极发展装配式建筑。争创国家海绵城市试点、省级节水型城市。增强全民节约意识、环保意识、生态意识。

（七）以共建共享为根本，不断提升幸福指数。

优先发展教育事业。实施第三期学前教育行动计划，促进学前教育普惠健康发展，解决好婴幼儿照护和儿童早期教育服务问题；统筹推进县域内城乡义务教育一体化发展，着力解决中小学生课外负担重、“择校热”、“大班额”等突出问题。加快建设东坡中学、永乐小学，改造实验中学、第五中学，切实解决滁城入学难问题。推进现代学徒制和学分制改革，加快建设职教强市。推进第二期特教行动计划。深化与安徽科技学院、滁州学院的双向互动，建立常态化、制度化的交流对接机制，不断扩大合作成果；推进职业技术学院、城市职业学院创建地方技能型高水平大学。

完善社会保障体系。打好民生保障战役，坚持就业优先战略和积极就业政策，鼓励创业带动就业，新增城镇就业4万人。加强职业技能培训，培育新型职业农民，注重解决结构性就业矛盾。实施“全民参保计划”。

推动健康滁州建设。加强基层医疗卫生服务体系和全科医生队伍建设，推动优质医疗资源有序有效下沉。鼓励社会办医，推动“互联网+”健康医疗，积极发展康体养生、健身休闲等新业态，推进医养结合。实施中西医结合医院、二院扩建工程。广泛开展全民健身活动，加强群众健身活动场地和设施建设，推进体育强市、体育强县创建工作。

促进文化事业发展。完善公共文化服务体系，活跃群众文化生活，创建第四批国家公共文化服务体系示范项目。市图书馆新馆建成开放，数字农家书屋加快建设。举办第七届中国农民歌会。落实好《滁州市非物质文化遗产保护条例》，加强文物保护利用和文化遗产保护传承，实施好琅琊山摩崖石刻及碑刻保护工程。注重挖掘欧阳修、王阳明、辛弃疾等名人文化，争创省级历史文化名城。讲好滁州故事，传播滁州声音。

深化平安滁州建设。打好平安建设战役，扎实开展社会治理创新行动，不断提升社会化、法治化、智能化、专业化水平。深入推进社会矛盾化解，妥善处理道路交通安全、问题楼盘处置、物业规范管理等方面问题。加强社会治安防控体系建设，实施滁城“雪亮工程”。促进大数据应用与社会治理深度融合。强化食品药品监管，争创全省食品药品安全示范市。推进安全生产“铸安”行动全覆盖，加强综合防灾减灾救灾能力和应急体系建设，坚决遏制重特大事故发生。

统筹发展其他事业。推动军民融合深度发展，加强国防动员和后备力量建设，巩固双拥成果。支持工会、共青团、妇联、文联、侨联、科协等群团工作，进一步加强外事、侨务、民族、宗教、统计、物价、质量、编制、档案、保密、人防、地震、气象、消防、老干部、地方志、红十字会等工作。

着力办好十件实事。今年市政府在滁城继续为市民办好十件实事：改造棚户区6500户；改造老旧小区35个、安置小区20个；迁建农贸市场1个、升级改造2个、治乱提标9个；完成18个社区办公用房达标工程；改造背街巷道49个；改造公厕74座、新建6座，改造垃圾中转站12座、新建3座；完成主城区水体环境治理5个；新增公办幼儿园3所、普惠性民办幼儿园5所；新建社会公共停车场2个；实施老年人高龄津贴提标以及免费乘坐城市公交车扩面至滁城65周岁以上老年人和所有持证残疾人。

（八）以人民满意为追求，切实加强自身建设。

打造学习型政府。扎实开展“不忘初心、牢记使命”主题教育，把学习宣传贯彻党的十九大精神作为首要政治任务，坚定用习近平新时代中国特色社会主义思想武装头脑、指导实践、推动工作，提高政治站位和政治觉悟，强化政治定力和政治担当，切实增强“八种本领”。

打造创新型政府。坚持“优”处着眼，用创先争优立标杆，用创新创造塑精品，用担当作为攀高峰，做到有冠必夺、有先必争、有旗必扛。坚持“高”处着力，把高标准、高质量、高水平作为事业追求，干到一流、干到最好、干到极致。坚持“新”处着手，把新理念、新机制、新方法作为推进措施，努力突破重点、攻克难点，实现新目标、创造新业绩、追求新跨越。

打造法治型政府。严格依法全面履行政府职能，做到法定职责必须为、法无授权不可为。依法接受市人大及其常委会的监督，自觉接受市政协的民主监督，主动接受社会和舆论监督，认真听取人大代表、政协委员、民主党派、工商联、无党派人士和人民团体的意见建议。认真办理人大议案、建议和政协提案。坚持依法民主科学决策，完善重大事项公众参与、专家论证、合法性审查等制度。加强政府信用建设，坚决履约守信，言必信、诺必践、行必果。

打造实干型政府。政府系统及其工作人员要干字当头，干在实处、走在前列。要敢于担当抓落实，不驰于空想、不骛于虚声，勇于挑最重的担子，敢于啃最硬的骨头，善于接最烫的山芋，做新时代的坚定者、奋进者、搏击者，不做犹豫者、懈怠者、畏难者。要雷厉风行抓落实，立说立行、紧张快干，用真抓的实劲、敢抓的狠劲、善抓的巧劲、常抓的韧劲，一任接着一任干，一张蓝图绘到底。要优化机制抓落实，牢固树立一盘棋思想，相互支持，相互配合，相互补台，共同营造干事创业的良好环境。

打造服务型政府。进一步转变政府职能，实现“双随机一公开”监管全覆盖，积极推进行政审批、公共资源交易和服务事项网上办理，努力构建便民高效的“互联网+政务服务”新模式。提升政府执行力，定期开展网络问政、行风评议、明查暗访等活动，坚决纠正不作为、乱作为，坚决克服懒政、怠政，坚决惩处失职、渎职，推动政府工作提速提效。

打造清廉型政府。把纪律和规矩挺在前面，深入推进党风廉政建设和反腐败斗争，全面落实“两个责任”，认真履行“一岗双责”。纠正“四风”不止步，着力推动中央八项规定精神落地生根。强化审计监督，管好公务人员、公共资源、公共权力、公共资金，着力营造干部清正、政府清廉、政治清明的良好局面。

各位代表、同志们，雄关漫道真如铁，而今迈步从头越。站在新时代新的历史起点上，滁州前程灿烂，我们重任在肩。让我们紧密团结在以习近平同志为核心的党中央周围，在省委、省政府和市委的坚强领导下，秉承滁州人民敢为天下先的勇气和担当，汇聚450万皖东儿女的智慧和力量，奋力拼搏、扎实苦干，为冲刺总量全省第三、决胜全面建成小康社会、开创现代化新滁州建设新局面而不懈奋斗！
